# Modelling and Evaluation of the Absorption of the 866 MHz Electromagnetic Field in Humans Exposed near to Fixed I-RFID Readers Used in Medical RTLS or to Monitor PPE

**DOI:** 10.3390/s21124251

**Published:** 2021-06-21

**Authors:** Patryk Zradziński, Jolanta Karpowicz, Krzysztof Gryz, Grzegorz Owczarek, Victoria Ramos

**Affiliations:** 1 Laboratory of Electromagnetic Hazards, Central Institute for Labour Protection–National Research Institute (CIOP-PIB), Czerniakowska 16, 00-701 Warszawa, Poland; jokar@ciop.pl (J.K.); krgry@ciop.pl (K.G.); 2 Eye and Face Protection Laboratory, Central Institute for Labour Protection–National Research Institute (CIOP-PIB), Czerniakowska 16, 00-701 Warszawa, Poland; growc@ciop.lodz.pl; 3 Telemedicine and e-Health Research Unit, Instituto de Salud Carlos III, Avda. Monforte de Lemos, 5, 28029 Madrid, Spain; vramos@isciii.es

**Keywords:** biomedical engineering, environmental engineering, numerical simulations, occupational exposure, public health, specific energy absorption rate (SAR), RadioFrequency IDentification (RFID), Real-Time Location Systems (RTLS), Internet of Things (IoT), Personal Protective Equipment (PPE)

## Abstract

The aim of this study was to model and evaluate the Specific Energy Absorption Rate (SAR) values in humans in proximity to fixed multi-antenna I-RFID readers of passive tags under various scenarios mimicking exposure when they are incorporated in Real-Time Location Systems (RTLS), or used to monitor Personal Protective Equipment (PPE). The sources of the electromagnetic field (EMF) in the modelled readers were rectangular microstrip antennas at a resonance frequency in free space of 866 MHz from the ultra-high frequency (UHF) RFID frequency range of 865–868 MHz. The obtained results of numerical modelling showed that the SAR values in the body 5 cm away from the UHF RFID readers need consideration with respect to exposure limits set by international guidelines to prevent adverse thermal effects of exposure to EMF: when the effective radiated power exceeds 5.5 W with respect to the general public/unrestricted environments exposure limits, and with respect to occupational/restricted environments exposure limits, when the effective radiated power exceeds 27.5 W.

## 1. Introduction

### 1.1. The Internet of Things

Communication technologies have been used in health protection since the 1990s [1]. The term “the Internet of Things” (IoT) was introduced in 1999 to express the concept in which physical objects could directly or indirectly obtain, store, send, or process data, mostly via computer networks [2]. After more than 20 years of continuous development, IoT is now the basic platform on which the concept of Industry 4.0 and health monitoring systems (Healthcare 4.0) are developing [3]. One of the widely used communication technologies that may be incorporated into the IoT concept is the RadioFrequency IDentification (RFID) system [4,5,6,7,8,9]. The combination of these is known as IoT RFID (I-RFID) [10]. These systems have also been widely used for many years in applications incorporating humans wearing active or passive components of an I-RFID network.

To date, no conclusive definition of IoT has been widely adopted, despite a vast body of publications on the subject [11]. One way to describe the IoT concept is to acknowledge the great proliferation of interconnected smart products with advancements in the field of sensor technology and internet connectivity [12]. In this approach, the internet has been extended to encompass physical objects capable of communicating with each other. Thus, the internet as a technology is thought to form a sphere that could also accommodate a multiplicity of smart devices, and as such it is termed the Internet of Things. In another approach, the IoT concept is grounded in machines and devices equipped with sensors and actuators for carrying out actions [13]. In this context, these interconnected physical objects delineate areas for internet activity, such as collecting data, remote monitoring, decision-making algorithms, process optimisation, etc. [14].

The advantages and possibilities of IoT have meant that, among other things, it is used in newer and newer applications. Some examples of this include: Real-Time Location Systems (RTLS), applications to monitor and control the allowed duration of use or technical condition of monitored Personal Protective Equipment (PPE) [15], and recently, applications to identify people infected with the SARS-CoV-2 coronavirus [13,15,16,17,18,19]. The electromagnetic safety aspects of the use of medical RTLS and PPE applications are presented in the further part of the article, because their functioning usually involve humans being in the vicinity of active elements of the systems, which work by emitting an electromagnetic field (EMF).

### 1.2. Examples of Real-Time Location Systems (RTLS) and Personal Protective Equipment (PPE) Monitoring Systems

**Figure 1:**
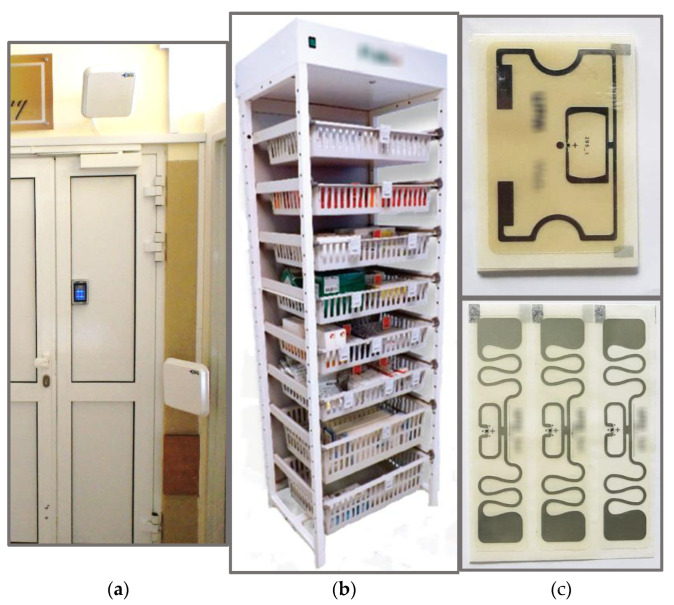
Examples of RFID systems in RTLS: (**a**) UHF RFID antennas at the entrance door to an operating theatre; (**b**) a shelf for biological materials and pharmaceuticals storing in medical centres and (**c**) examples of RFID passive tags.

RTLS are used for identifying, monitoring and tracking objects within indoor or confined environments (e.g., tracking devices, things or people in factories, warehouses or offices, and even tracking patients, biological materials and pharmaceuticals in medical centres) [17]. All RTLS applications consist of following components: transponders, receivers, and software to analyse and interpret the data exchange between them. The complexity of the system and kind of the application determinates the technology, and thus the hardware and software required to develop the best-suited RTLS. One of the most widely used communication technologies that may be incorporated in RTLS is the RFID system [4,5,6,7,8,9]. This identification technology uses radio waves (electromagnetic non-ionising radiation) to transmit data and, when required by the use of passive tags, also to wirelessly power an electronic circuit attached to objects covered by the application. The technology frequently implanted in RTLS systems is UHF (Ultra-High Frequency) RFID, which emits an EMF at a frequency from the bands 865–868 MHz and 915–921 MHz for typically passive tags, and 433 MHz for active tags. In RTLS based on UHF RFID technology, the transponders are tags (passive or active) attached to persons or objects, and the receivers are RFID readers. These readers may consist of one to more than 10 antennas and may be fixed to the walls, ceiling or mounted in dedicated frames, for example in the form of a panel (similar to a gate panel used in electronic article surveillance (EAS) systems, including, for example, three antennas in a single panel, or a shelf/cabinet with pharmaceuticals or biological samples) as shown at Figure 1. 

The maximum reading range (i.e., the maximum distance where tags efficiently communicate with the reader) in systems using UHF RFID passive tags depends on the power emitted from the reader antenna, the tag sensitivity and the conditions of wave propagation [4]. Typically, it does not exceed 20 m, though the majority of applications operate at a much shorter reading range. Depending on the sensitivity of the passive tags used, UHF RFID readers used in such systems have to operate for example at an output power of around 3–30 W for a 25 m reading range, 0.2–2.0 W for a 5 m reading range and 0.01–0.10 W for a 1 m reading range [4,20]. Passive tags are more frequently used than active tags, due to their significantly lower costs and operation time not being limited by the life of batteries, which are required to power active tags.

Systems managing monitored PPE usually use the same UHF RFID technology as RTLS. PPE also usually uses passive RFID tags, operating at a typical reading range not exceeding several metres [15]. Depending on the sensitivity of the passive tags used, UHF RFID readers used in such systems have to operate at an output power of around 0.4–4.0 W for a 7 m reading range, 0.12–1.2 W for a 4 m reading range and 0.03–0.30 W for a 2 m reading range [4,15,19].

**Figure 2:**
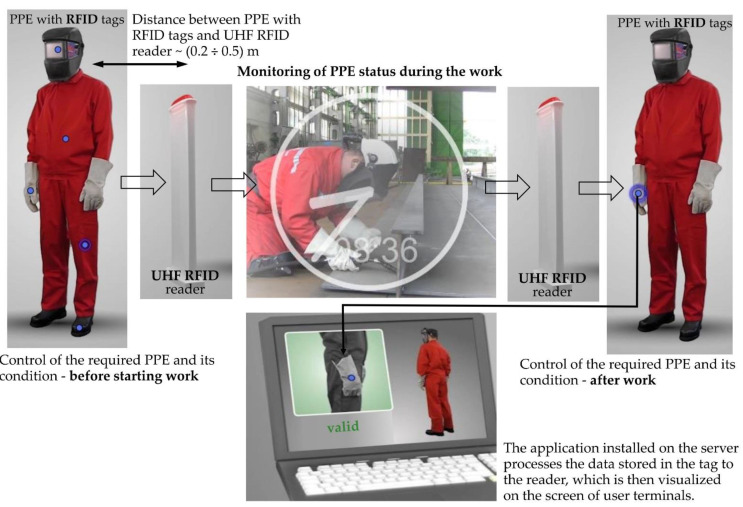
The principle of operating the system of automatic identification and management of PPE in the workplace.

An example of this is the system of identifying and managing personal protective equipment, taking into account two levels of monitoring the technical condition of PPE: Level 1—storage of PPE and Level 2-maintenance and use of PPE [19]. The system consists of: electronic RFID passive tags, antennas (a UHF RFID reader), a server operating with core software, a database of PPE characteristics and terminals (user computers) providing real-time communication for PPE users (Figure 2) [15,19]. The RFID reader communicates wirelessly with the electronic tag attached to the PPE (e.g., protective gloves, helmets, clothes or shoes), updating the database on the server computer. The computer application installed on the server processes this data and distributes relevant communications to terminals. Using the system described above, PPE is monitored before starting work and periodically (as specified in the PPE manufacturer’s instructions). The procedure enables the use of PPE to be controlled, and detects any cases of use of the PPE longer than its “allowed duration of use”, as defined by relevant technical guidelines. The workers using monitored PPE may pass through the UHF RFID reader gates several times during one working day, in a number of zones around the workplace [19]. The PPE monitoring systems considered here were developed for hazardous industrial environments, but are spreading also to other applications, including healthcare environments.

In short, in both examples mentioned above, the tracking of objects, such as tools, pharmaceuticals and biological samples monitored by RTLS, and manually managed by workers, or of other people, for example patients monitored by RTLS or users of labelled PPE, requires people to approach directly to RFID readers, and thus be exposed to the EMF emitted there.

The electric field strength in the vicinity of UHF RFID readers mainly depends on the required reading range and the sensitivity of the read passive tags, which together define the power radiated by the reader necessary for a particular system to work efficiently. The minimum electric field strength required at the tag location to read a passive tag typically ranges from 0.6 V/m for the latest, more sensitive tags, but up to 2 V/m for older, less sensitive passive tags [4]. For example, electric field strengths at a radiated power of 1 W from a single antenna UHF RFID reader are approximately 50 V/m at 10 cm, 12 V/m at 50 cm, 7 V/m at 100 cm and 2 V/m at a 300 cm distance from the reader centre [4], allowing less sensitive passive tags to be read in objects located up to 300 cm away from the reader.

### 1.3. The Metrics of Exposure to Radiofrequency EMF

Extensive studies on the biophysical and biomedical effects caused in humans by radiofrequency EMF exposure have nearly the same duration of history as the use of such EMF in radio communication and other applications opened by experiments of Nicola Tesla and Guillermo Marconi in the very last years of 19th century. The scientific outcomes from such studies span a very-broad variety of beneficial and adverse effects of EMF influence—whether well documented or still hypothesised—summarised in highly reputable monographs, review reports and pooled analyses [21,22,23]. The biophysical effects of EMF exposure are based mainly on the electric and magnetic fields induced inside body structures and are highly dependent on the EMF frequency and characteristic of exposure in time and space, as well as the health status of the exposed person. The studied adverse health effects of EMF exposure include carcinogenic hazards, as summarised by IARC in a monograph providing the rationale for 2B classification of radiofrequency EMF exposure [22]. Taking into account the EMF exposure of all modern society, as well as scientific evidence supporting hypotheses about possible links between EMF exposure and various health hazards, reports summarising the research requirements in the area of health hazards from environmental factors still highlight the need for better organised and more systematic studies on EMF exposure and its possible health consequences [23,24,25]. 

Contrary to the broad variety of biophysical and biomedical studies on EMF exposure effects, the formal (legal-based) evaluations of EMF safety near to radiofrequency EMF emitting devices tend to widely accept simplified biophysical considerations for the safety requirements, known as “minimum safety requirements”. These are based on the use of parameters characterising electromagnetic absorption in the body, evaluated by numerical simulations in relevant exposure scenarios, where realistic models of EMF sources, the human body and the surrounding environment are mimicked.

For the frequency range including EMF emitted from the considered RFID systems, the international safety guidelines are based on the outcome of studies on the thermal effects in the exposed body and direct biophysical mechanisms of the thermal impact from the human body exposure to high-frequency EMF or microwaves (considered to be a dominant effect of the direct biophysical influence of EMF at frequencies exceeding 10 MHz). This effect is related to tissue heating and the thermal load of the whole body due to the electromagnetic energy absorbed [26,27,28,29]. 

The worldwide accepted metric used to evaluate the direct thermal effects relating to exposure to EMF in the frequency range 100 kHz–6 GHz is the Specific Energy Absorption Rate (SAR), expressed in watts per kilogram, W/kg, and usually evaluated by numerical simulations using complex numerical models of the human body. International guidelines provide SAR limits split into three categories: whole-body averaged SAR (should be averaged over 6 min according to Directive 2013/35/EU and ICNIRP 1998; or over 30 min according to ICNIRP 2020 and IEEE C95.1-2019); or local head and torso SAR and local limb SAR (averaged over 10 g cubic mass, and over 6 min) (Table 1) [26,27,28,29]. SAR limits published by ICNIRP 1998 were used in various legislative documents dealing with the formal requirements regarding protection against electromagnetic hazards, for example in the binding European Directive 2013/35/EU [20,28]. Higher limits provided for occupational exposure (recognised also for exposure of a person in a restricted environment) are applicable only in the work environment where relevant protection measures are applied, workers are aware of exposure parameters and related health and safety hazards, etc. [28,29].

For the evaluation of EMF exposure in the far-field region (at least one wavelength distance from the EMF source), it is possible to apply also secondary, operational, exposure limits derived from the SAR limits and expressed in the values of electric field strength (E, V/m) and magnetic field strength (H, A/m). However, in the case of a close distance between the EMF source and the exposed body, a direct use of SAR evaluation is recommended by international safety guidelines and legislation [27,28].

There is a scarcity of published works on the evaluation of EMF exposure caused by the use of RFID systems. Many of them concern only technical aspects related to the implementation of RFID systems (often using frequencies, devices and applications other than as presented in this study) in various sectors of the economy and in life (examples are presented in the previous sections). Only a few publications concern EMF parameters in the vicinity of such devices, and even fewer are related to SAR simulations. The previous publications of the authors concerned an analysis of SAR: during exposure to HF RFID readers (13.56 MHz) and during the use of handheld UHF RFID readers equipped with a single antenna [4,30]. The results of this study are novel with respect to the frequency and structure of the EMF source and exposure scenarios, as no similar published works by other authors have been found. The outcome of the analysis at a lower EMF frequency and at more localised exposure (caused by a single antenna of the hand-held RFID reader considered in earlier article) is not relevant to the multi-antenna readers considered here, because the ratio of SAR values to the level of EMF exposure is sensitive to EMF frequency and spatial distribution.

Methods of SAR modelling have become standardised research procedures, though the analysis of SAR in realistic cases using relevant models mimicking exposure scenarios is still the subject of research and open discussion on the differences of EMF influence in cases of EMF exposure recognised as caused by “the intended use”, “the worst-case exposure scenario” or “reasonably foreseeable use” [31,32].

### 1.4. The Aim

The aim of this study was to evaluate SAR values in the body of a person present near to fixed multi-antenna readers of UHF RFID passive tags to test the hypothesis that the use of modelled systems does not cause EMF exposure exceeding relevant limits provided for the evaluation of exposure of the general public/a person in unrestricted environments. SAR values were evaluated under various exposure scenarios, derived to mimic EMF exposure related to particular applications of IoT systems, for example, by the use of medical RTLS or monitored PPE usage systems, operating at 866 MHz. The considered applications of RFID systems cause human EMF exposure because of the manual management of tracked objects in a healthcare environment, or the use of tracked PPE, which is specific compared, for example, to industrial applications where many RFID systems are managed by the automatic processing of tracked objects, and in this case EMF exposure in humans is on a much lower scale.

## 2. Materials and Methods

### 2.1. Numerical Model of the EMF Source

The modelled EMF source was a rectangular microstrip patch antenna designed to operate at a frequency of 866 MHz to support the frequency range of UHF RFID applications used in Europe (chosen as the centre frequency in the most commonly used frequency range 865–868 MHz). An antenna with dimensions of 99.6 × 122.5 mm was placed on substrate and ground layers with dimensions of 131.5 × 138.3 mm, for more details see [4]. Copper with a thickness of 0.035 mm, an electric conductivity of 5.813 × 10^7^ S/m and a relative permittivity of 1 was used for the antenna; Rogers RO3003 with a thickness of 1.52 mm, an electric conductivity of 2.2 × 10^−4^ S/m and a relative permittivity of 2.92 was used as the substrate; ground with a thickness of 0.035 mm was modelled as PEC (perfect electric conductor). Elements related to the antenna housing, its fixation, gun handle, and monitor were not included in the numerical model.

The numerical model of the developed UHF RFID antenna was validated by comparing the electric field strength distributions near the antenna, obtained by numerical simulations, with measurements inside a semi-anechoic chamber; for more details see [4]. The differences in electric field strength values measured and numerically simulated were found to be less than 10%.

**Figure 3:**
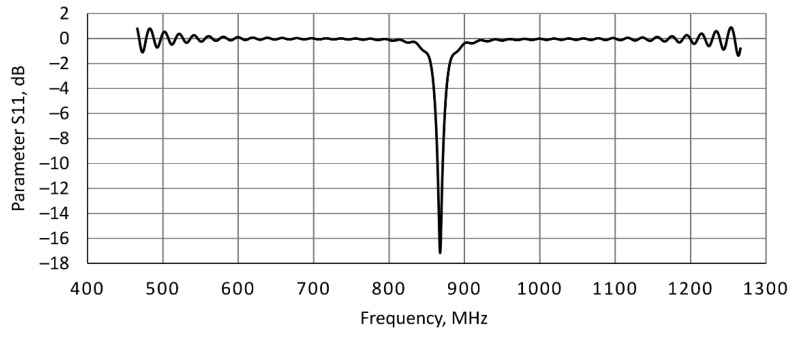
The S11 parameter determined using numerical simulations of the developed numerical model of the rectangular microstrip patch antenna used in the UHF RFID readers.

Figure 3 shows the S11 reflection coefficient parameter of the numerical model of the rectangular microstrip patch antenna used in the UHF RFID readers. The results of numerical simulations show that antenna was designed to operate at 866 MHz in the free space, to support the frequency range 865–868 MHz of UHF RFID applications–centre (resonance) frequency with an S11 parameter of −17.2 dB. The antenna gain was 5.35 dBi.

### 2.2. Exposure Scenarios

**Figure 4:**
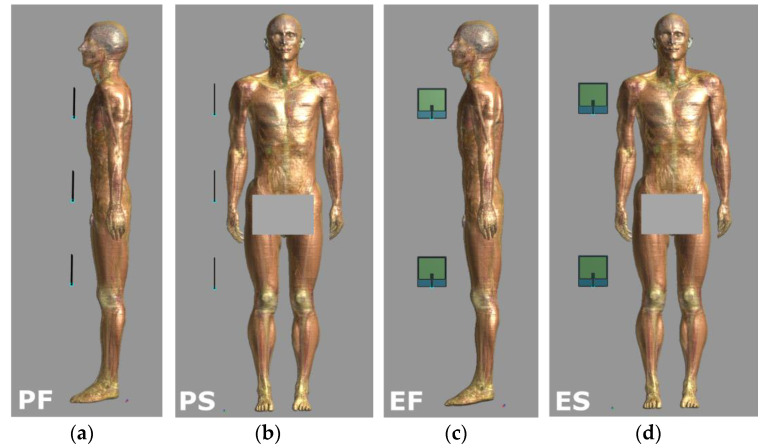
Exposure scenarios to EMF near UHFI-RFID multi-antennas readers: (**a**) three antennas plane in front of (PF) and (**b**) to the side of (PS); and (**c**) two antennas side-on in front of (EF) and (**d**) to the side of (ES) human body model.

The investigations covered exposure scenarios with various frequently used multi-antenna UHF RFID readers fixed to the walls or mounted in a dedicated frame in various locations against the nearby human body, located in a significantly varied, realistic standing position (Figure 4):

- Scenario PF—two or three antennas plane (P) located in front (F) of the human body;
- Scenario PS—two or three antennas plane (P) located to the side (S) of the human body;
- Scenario EF—two or three antennas side-on (E) located in front (F) of the human body;
- Scenario ES—two or three antennas side-on (E) located to the side (S) of the human body.

In all the exposure scenarios, the UHF RFID antennas were located at a distance of 5, 20 or 40 cm to the closest surface of the human body. The centres of the antennas and the main axis of the human body lay in one plane. In the exposure scenarios with three antennas the centre of the lower antenna was placed at a height of 626 mm, the middle antenna at a height of 1018 mm and the upper one at a height of 1410 mm. In exposure scenarios with two antennas the middle antenna was omitted. The variety of exposure scenarios were tested as required by European Directive 2014/53/EU (known as the RED Directive), to cover “all intended operating conditions” (distances of 20 cm and 40 cm) and “reasonably foreseeable conditions” (distance of 5 cm—the closest justifiable distance expected in real conditions of use of the UHF RFID system, designed without physical barriers or obstacles) [31]. 

### 2.3. Numerical Model of Human Body

An anatomical numerical male model—Duke—composed of over 300 tissues/organs, 177 cm tall, weighing 70.2 kg and with a BMI (body mass index) of 22.4, developed by the IT’IS (The Foundation for Research on Information Technologies in Society, Zurich, Switzerland), was used in the investigations [33]. It was chosen because its anthropometric data are close to the 50th percentile of male population data—data from ICRP 110 Adult Reference Computational Phantoms [34]. 

Dielectric parameters and densities of particular body tissues, of which the Duke numerical model is composed of 866 MHz frequency, were from the IT’IS Database for thermal and electromagnetic parameters of biological tissues [35].

### 2.4. Numerical Simulations

Numerical simulations were carried out using Sim4Life software (Zurich Med Tech, Zurich, Switzerland) using Single EM FDTD (finite difference time-domain) solver. The performed simulations were run with all the EMF sources considered in each exposure scenario (voltage edge sources) excited simultaneously. ABC (absorbing) boundary conditions were applied on the walls of the simulation domain. The simulation domain (except for its lower wall) was extended by 500 mm in each direction to avoid the influence on the considered EMF exposure from the boundaries. The finest resolution used in the investigations was 0.007 mm set for the antenna, and 2 mm set for the model of the human body (finer than 1/15 of the wavelength in tissues at 866 MHz). The uncertainty of numerical simulations (related to the models of field source and human body, type and location of the boundary conditions with respect to the model of the human body, position and the dielectric properties of the model of human body) was estimated as not exceeding ±25% (K = 1), within the range compliant with state-of-art in the field [36].

## 3. Results

**Figure 5:**
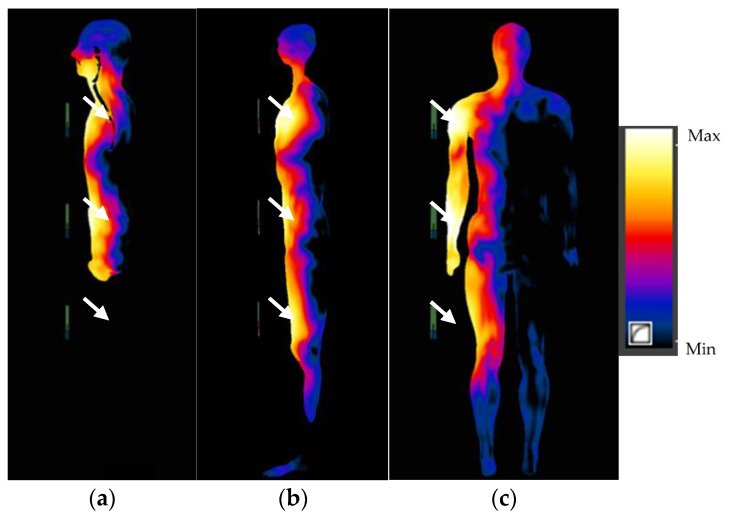
SAR distributions in the human body exposed to EMF near a UHF I-RFID three-antenna reader located 5 cm from the body: (**a**,**b**) in the body’s sagittal cross-sections through the body’s main axis and 6 cm to side (through the leg) in scenario PF and (**c**) in the body’s frontal cross-section in scenario PS; antennas marked with white arrows.

**Figure 6:**
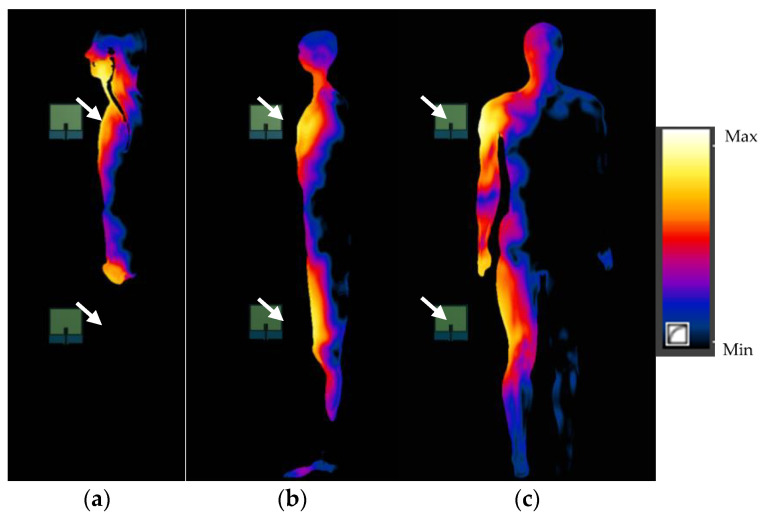
SAR distributions in the human body exposed to EMF near a UHF I-RFID two-antenna reader located 5 cm from the body: (**a**,**b**) in the body’s sagittal cross-sections through the body’s main axis and 6 cm to side (through the leg) in scenario EF and (**c**) in the body’s frontal cross-section in scenario ES; antennas marked with white arrows.

**Figure 7:**
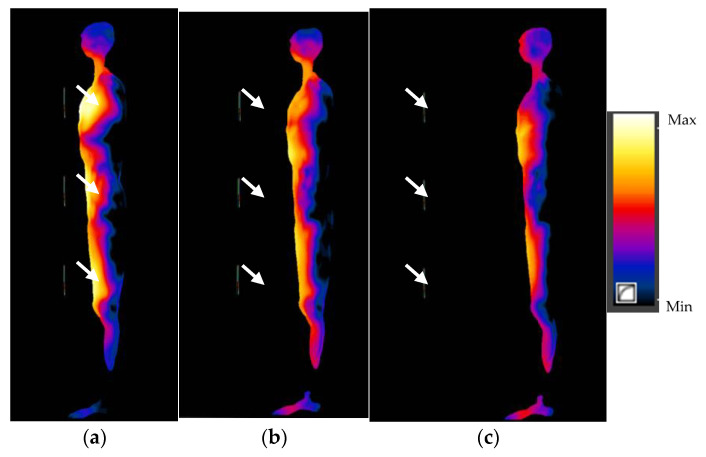
SAR distributions in scenario PF in the sagittal cross-section of the human body exposed to EMF near a three-antenna UHF I-RFID reader, located: (**a**) 5 cm; (**b**) 20 cm and (**c**) 40 cm away; antennas marked with white arrows.

The numerical simulations performed allowed the SAR values in anthropoidal human body models exposed near the multi-antenna fixed UHF I-RFID readers to be calculated. The distributions of SAR values in the human body exposed 5 cm away from antennas (in the body sagittal cross-section and in the body frontal cross-section in particular exposure scenarios) are shown in Figure 5 and Figure 6. To analyse the influence caused by the variations in distance from the reader antennas on the SAR distributions in the exposed human body, a comparison of SAR distribution in exposure scenario PF in the sagittal cross-section of body located at 5; 20 and 40 cm away from the three-antennas UHF I-RFID reader is shown in Figure 7.

Table 2 shows the results of the numerical simulations of SAR values related to exposure to EMF at 866 MHz near to a multi-antenna UHF I-RFID reader at an input power to each single antenna of 1 W, analysed with respect to continuous exposure–i.e., the worst-case exposure scenario with respect to the exposure duration and the rules of evaluating the thermal load from human exposure to EMF. In the case of a shorter duration of EMF exposure, SAR values are lower because they are set out to be time-averaged over six minutes for local SAR averaged over a 10 g mass of any continuous tissue (SAR10g), or over 30 min for SAR averaged over the entire exposed body (WBSAR).

The highest SAR values were found in exposure scenario PF (antennas plane located in front of the human body model)–0.231 W/kg for SAR10g and 0.012 W/kg for WBSAR in exposure involving a three-antenna reader, and 0.225 W/kg and 0.007 W/kg in exposure near a two-antenna reader, respectively. The WBSAR values obtained for exposure scenarios with a two-antenna reader were up to 40% lower than the values obtained for exposure scenarios with a three-antenna one. Additionally, SAR10g values up to eight-times (in scenarios with the two-antenna reader) and six-times (in scenarios with a three-antenna reader) higher were found for the reader located in front of the human body, compared to cases with the reader located to the side, and values up to twice as high for exposure cases with the human body located near antennas plane in comparison to antennas side-on. With increasing distance to antennas of the reader, SAR values decrease significantly: up to 2.8 times comparing the 20 cm distance to the 5 cm distance, and up to 10 times comparing the 40 cm distance to the 5 cm distance.

## 4. Discussion

The development of the broad use of I-RFID systems, exemplified by monitoring the location of the material, pharmaceuticals, healthcare personnel or patients in any healthcare environment or monitoring workers and PPE use in any work environment involves wireless communications system. This supposes the appearance of new sources of electromagnetic radiation and makes it necessary to analyse their influence on the electromagnetic environment.

Electromagnetic emissions and their possible direct or indirect effects on people’s safety is an issue rarely discussed in relation to IoT. A 2008 report from DG INFSO and EPoSS, “Internet of Things in 2020: a roadmap for the future” [37] did not mention it at all. No reference to the risks or concerns that EMF may raise was also found in newer subsequent official reports focused on IoT. These include: (1) “Internet of Things–From Research and Innovation to Market Deployment”, published in 2014, which describes the commercial exploitation of many of the IoT research and innovation lines that had been developing up to that moment, as well as its proposals for the future [38], or (2) the first joint text on the Internet of Things approved by the European authorities on 16 September 2014–specifically “Opinion 8/2014 on Recent Developments on the Internet of Things” [39]. Although Opinion 8/2014 raises many scenarios considering electromagnetic emissions—considering wearables, biometrics and domestic—only a few refer to these electromagnetic scenarios [40] and the majority focus exclusively on the information-related vulnerability of users such as the loss of their information, malware, unauthorised access to personal data, the intrusive use of portable devices or illegal surveillance [41]. Since then, the European Commission has continued to promote and finance lines of research on IoT, mainly for the development of the so-called digital single market, with new documents and standards [42] promoting this technological gamble, but again without referring to the energy of electromagnetic emissions. Broader international standardisation also covers IoT development, for example the one provided by the International Telecommunication Union (ITU) [43] illustrates the evolution of the IoT towards the Internet of Everything.

However, it must be raised that adequate protection of European citizens against excessive influence from electromagnetic radiation in public and in workplace environments is required by relevant legislation characterised in Section 1.3. Consequently, it is necessary to verify that the EMF intensity does not exceed the recommended exposure limits and to guarantee the safety of humans. It is also necessary to maintain the electromagnetic compatibility requirements established by the regulations in view of the possible coexistence between IoT devices and implantable devices, which may require the application of protection measures to ensure their lower exposure than allowed for the general population [30,44,45]. The probability of the use of medical implants is the highest in healthcare environments, where their users need to receive various services (as patients), and sometimes also healthcare personnel are using implants.

The appearance of these new sources of radiation requires a study of the current exposure situation in a number of areas and is characterised by the use of devices, wireless communication terminals, and electrical appliances whose operation involves the generation of EMF. It will be necessary to analyse its influence on the electromagnetic environment due to the existence of other devices and communications networks that also work wirelessly. The measurement and control of EMF are increasingly necessary, not only in healthcare environments, but also in other sensitive environments with new wireless communication systems where a large amount of electrical and wireless equipment coexist. It is necessary to check that the EMF intensity does not exceed the recommended limits, as well as ensuring the safety of humans according to the relevant guidelines and requirements [22]. 

All of these aspects are very important and indicate the directions of further research on electromagnetic hazards from IoT devices. However, this study was focused on an analysis of the direct biophysical effects of exposure to EMF characterised by SAR values in the body (i.e., parameters characterising the thermal load in the body exposed to radiofrequency EMF) of a person present near to fixed UHF I-RFID readers of passive tags, and its compliance assessment against limits provided by relevant guidelines.

According to ETSI EN 302-208 V3.3.1 (2020-08), being a harmonised standard with Directive 2014/53/EU (recognised as the RED Directive) for RFID equipment operating in the 865–868 MHz and 915–921 MHz bands, their use does not require special permission when the effective radiated power (ERP) from the antenna does not exceed 2 W or 4 W, respectively [31,46]. Available on the market RFID devices equipped with more powerful antennas should be used only when adequate administrative permission has been obtained. The results of this study showed that SAR values in the body exposed to EMF at 5 cm away from multi-antenna UHF I-RFID readers may exceed the limits of SAR10g in the torso and head, regarding the general public exposure (2 W/kg), when the ERP from each antenna exceeds 15 W; and limits of WBSAR (0.08 W/kg) when the ERP from each antenna exceeds 11 W (Table 3 and Table 4). 

However, taking into account also the expanded uncertainty of the discussed numerical simulations, the WBSAR and SAR10g limits may be exceeded in the considered exposure scenario at the EMF emission levels from each antenna of 5.5 and 7.5 W, respectively. This emission level must be used in I-RFID systems operating with passive tags of various sensitivities, when the required maximum reading range reaches 6–24 m [4]. From the other point of view, the results of this study confirmed also that the analysed SAR parameters do not exceed mentioned limits regarding general public exposure near RFID devices designed similarly to considered one and with EMF emission at level which does not require special permission (the emission from each antenna <2 W).

The ratio of input power to each antenna to ERP emitted from each antenna is not constant. The highest ratio was found in exposure scenario PF (antennas plane located in front of the human body model) at a distance of 5 cm. The reason for this is that the human body (the largest surface parallel to the antenna plane) significantly affects the performance of the antenna located nearby.

It must be emphasised that the spatial distribution of EMF absorption inside a body is a function of the affecting EMF frequency, polarisation, spatial distribution, as well as body posture and electric grounding (or insulating) to conductive objects in the vicinity. The consequence of this is a highly varying ratio between the values of local and body averaged SAR. When the limits set for local SAR in the head and torso are 25-times higher than the limits for the whole body average, any evaluation of EMF exposure compliance needs to consider both kinds of limits in all cases of exposure characterised by the local to whole-body average SAR ratio (L2ASARR) exceeding 25 (usually in cases of highly localised exposure), or when the considered set of exposure scenarios show no correlation between local and averaged SAR values. On the other hand, for exposure scenarios in which local and average SAR values are correlated, or where L2ASARR is significantly lower than 25 in head and torso, and lower than 50 in limbs, the evaluation of EMF exposure compliance with considered EMF exposure limits may be sufficiently performed using just a whole-body average SAR–which makes this evaluation much easier, even achievable without sophisticated numerical simulations.

No correlation between WBSAR and SAR10g values for investigated exposure scenarios was found. The highest value, 32, of L2ASARR was found in exposure scenario PF with two antennas at a distance of 5 cm. For other exposure scenarios these ratios are significantly below 25. Conclusion from this is that in considered exposure scenarios, the compliance assessment with limits can be sufficiently done based on the WBSAR only, which is easier to estimate than the SAR10g. The obtained results indicate that such an assessment is sufficient in the case of exposure near to UHF I-RFID systems equipped with at least three antennas (even when they are located 5 cm from the human body) or near to systems equipped with one or two antennas at distances longer than 20 cm. 

According to European requirements, such as under EN 60601-1-2 (the harmonised standard for medical devices drafted in support of Council Directive 93/42/EEC, contained in and repealed by Regulation 2017/745 of the European Parliament and of the Council), in order to be released onto the European Union market, the functioning of medical devices in professional or home healthcare environments should be resistant (immune) to the influence of an electric field of the frequency range (80–2700) MHz with a strength of up to 3 V/m or 10 V/m, respectively [45]. Taking 10 V/m into account, I-RFID systems should not be used in places accessible to users of active medical implants (containing electronic circuits) at distances smaller than 1.2, 0.7 and 0.5 m, at EMF emission levels (ERP) of 5.5, 2 and 1 W from each antenna, respectively.

It must also be stated that RFID tags also emit EMF when communicating with the RFID reader, and the question on the EMF exposure of users of wearable elements of I-RFID systems may also be addressed. Since the emission from passive RFID tags of wearable elements of I-RFID system (when communicating with the RFID reader) is very low (significantly less than 1 mW) it is expected that effects of EMF exposure from such emissions are negligible compared to the considered exposure levels compliant with mentioned SAR10g limits [5].

## 5. Conclusions

By means of the numerical simulations performed, this work calculated SAR values in anthropoidal human body models exposed near to multi-antenna fixed UHF I-RFID readers in exposure scenarios realistic with respect to the use of such systems in the health care environment or for monitoring PPE use. 

The main contribution of this work is that the absorption in the human body of EMF emitted continuously by a multi-antenna UHF I-RFID reader incorporated in an RTLS or PPE monitoring system may have a significant influence on humans when the ERP from each antenna exceeds 5.5 W (the use of devices available on the market and emitting this level of power needs an appropriate administrative permission–ETSI EN 302-208 V3.3.1 (2020-08)) [46]. 

It also should be pointed out that EMF exposure from UHF I-RFID systems equipped with at least three antennas (even when they are located 5 cm from the human body) can be regarded as exposure case for which the compliance assessment with SAR limits can be done based on the WBSAR evolution only, which is easier to estimate than when both WBSAR and SAR10g need consideration.

In this work, the main contribution concerned multi-antenna UHF I-RFID readers as used in RTLS in medical centres, or PPE in industrial plants, but similar devices are widely useful in many other businesses, such as shops, warehouses, libraries and so on, and the EMF in their vicinity will be similar to that presented in our study.

Further research on protective actions (e.g., the physical distance between the UHF I-RFID antennas and the human body), taking into account anatomical variabilities between various humans and the vulnerable population of medical implant users. Moreover, future works should extend the analysis of measures of EMF exposure effects (SAR values) from exposure near RFID readers using various antenna configurations (new applications), the use of active RFID tags (cards/labels) working in other frequency bands (e.g., 433 MHz) and the expected wide use of wearable devices, sensors and/or actuators used in various sectors of the economy and life. The human body model used in this work reproduces the permittivity (dielectric constant, conductivity) of a wide variety of body tissues in the 866 MHz frequency band. It should be highlighted that the main benefit of this simulation is that the used dielectric properties of the tissue can allow the testing of safety of various future wireless devices, such as those of I-RFID, the Internet of Medical Things (IoMT) or bandwidths like those used in the new generation of radiocommunication networks (e.g., known as 5G mobile communications networks) before they become available on the market.

## Figures and Tables

**Table 1:** SAR limits applicable for evaluating exposure to EMF at 865–868 MHz and 915–921 MHz frequencies used by UHF RFID systems.

Exposure Scenario	Basic Restrictions/Dosimetric Reference Limits			EMF Frequency
	Whole-Body Average WBSAR, W/kg	Local Head/Torso SAR10g, W/kg	Local Limb SAR10g, W/kg	
Occupational/Person in restricted environments	0.4	10	20	100 kHz to 6 GHz (ICNIRP 2020, IEEE 2019) 100 kHz to 10 GHz (ICNIRP 1998) 6 to 300 GHz (WBSAR only, ICNIRP 2020)
General public/Person in unrestricted environments	0.08	2	4	

Note: 1—WBSAR values are to be averaged over the entire body and over a 30 min period (ICNIRP 2020, IEEE C95.1-2019) or a 6 min period (ICNIRP 1998); 2—SAR10g values are to be averaged over a 10 g cubic mass and over a 6 min period (ICNIRP 1998, 2020 and IEEE C.95.1-2019).

**Table 2:** SAR values in the human body model Duke for various exposure scenarios, at 5, 20 and 40 cm to UHF RFID antennas for an input power to each antenna of 1 W @ 866 MHz.

Exposure Scenario ^1^	WBSAR ^2^, W/kg			SAR10g ^3^, W/kg		
	5 cm	20 cm	40 cm	5 cm	20 cm	40 cm
2 antennas, PF	0.007	0.005	0.004	0.225	0.079	0.022
3 antennas, PF	**0.012**	0.008	0.006	**0.231**	0.113	0.053
2 antennas, PS	0.006	0.004	0.003	0.027	0.023	0.011
3 antennas, PS	0.010	0.007	0.005	0.039	0.032	0.027
2 antennas, EF	0.006	0.005	0.004	0.092	0.052	0.018
3 antennas, EF	0.010	0.008	0.006	0.112	0.063	0.033
2 antennas, ES	0.005	0.004	0.003	0.017	0.014	0.008
3 antennas, ES	0.008	0.006	0.005	0.022	0.019	0.015

^1^ Antennas plane (P) or side-on (E), located in front (F) of or to the side (S) of the human body; ^2^ WBSAR–SAR evaluated as averaged over the entire exposed body; ^3^ SAR10g–maximum local SAR averaged over a 10 g mass of any continuous tissue in the head and torso; the highest values in bold.

**Table 3:** The level of EMF emissions from UHF I-RFID readers at 866 MHz, causing WBSAR values in the human body model Duke equal to the general public limits in the considered exposure scenarios (SAR evaluated as averaged over the entire exposed body).

Exposure Scenario ^1^ at WBSAR = 0.08 W/kg	Input Power to Each Antenna, W			ERP ^2^ Emitted from Each Antenna, W		
	5 cm	20 cm	40 cm	5 cm	20 cm	40 cm
2 antennas, PF	11	15	19	22	26	28
3 antennas, PF	**6.9**	10	13	13	17	19
2 antennas, PS	14	18	22	16	22	31
3 antennas, PS	8.4	12	15	**11**	15	22
2 antennas, EF	12	17	20	19	22	25
3 antennas, EF	8.3	11	14	14	15	19
2 antennas, ES	16	21	24	20	26	31
3 antennas, ES	10	14	16	15	20	23

^1^ Antennas plane (P) or side-on (E), located in front (F) of or to the side (S) of the human body; ^2^ ERP—effective radiated power; the lower values in bold.

**Table 4:** The level of EMF emission from UHF I-RFID readers at 866 MHz, causing SAR10g values in the human body model Duke equal to the general public limits in the considered exposure scenarios (maximum local SAR averaged over a 10 g mass).

Exposure Scenario ^1^ at SAR10g = 2 W/kg	Input Power to each Antenna, W			ERP ^2^ Emitted from each Antenna, W		
	5 cm	20 cm	40 cm	5 cm	20 cm	40 cm
2 antennas, PF	**8.9**	25	90	18	44	120
3 antennas, PF	**8.7**	18	38	**15**	29	55
2 antennas, PS	73	88	190	89	110	270
3 antennas, PS	52	62	75	66	78	110
2 antennas, EF	22	39	110	33	51	140
3 antennas, EF	18	32	60	30	46	83
2 antennas, ES	120	140	250	150	180	320
3 antennas, ES	92	110	130	130	150	190

^1^ Antennas plane (P) or side-on (E), located in front (F) of or to the side (S) of the human body; ^2^ ERP–effective radiated power; the lower values in bold.

## Data Availability

Not applicable.
